# Supplementary material for: DNA nano-pocket for ultra-selective uranyl extraction from seawater
Source: Nat Commun. 2020 Nov 11;11:5708. doi: 10.1038/s41467-020-19419-z (PMC7659010; doi:10.1038/s41467-020-19419-z)
Supplement: Supplementary file 1 — Supplementary Information [file 41467_2020_19419_MOESM1_ESM.pdf]

Supplementary Information

DNA nano-pocket for ultra-selective uranyl extraction from seawater

Yihui Yuan†, Tingting Liu†, Juanxiu Xiao, Qiuhan Yu, Lijuan Feng, Biye Niu, Shiwei Feng, Jiacheng Zhang, Ning Wang*

Supplementary Figures

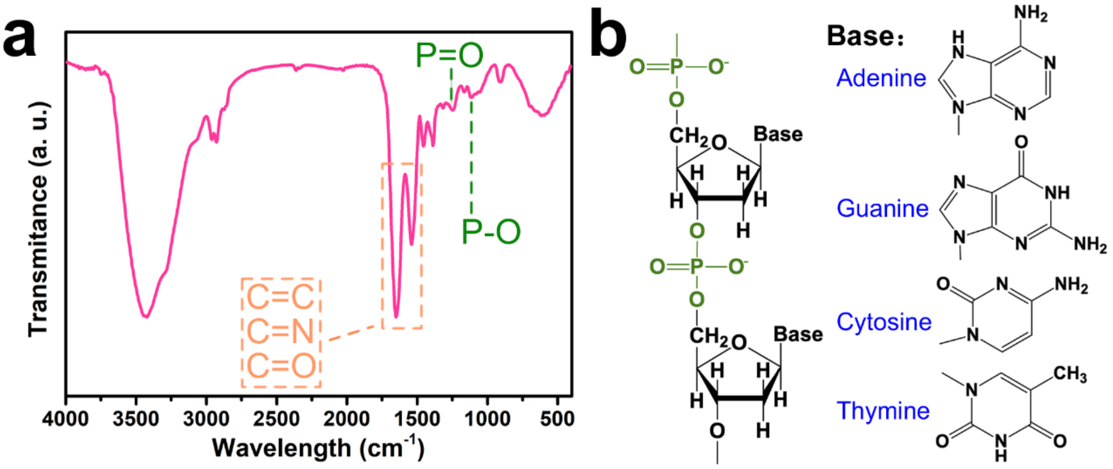

Supplementary Figure 1 Chemical composition of hydrogel DNA-UEH. **a** FTIR analysis of the hydrogel DNA-UEH. **b** Molecular structures of the component of the DNA molecular.

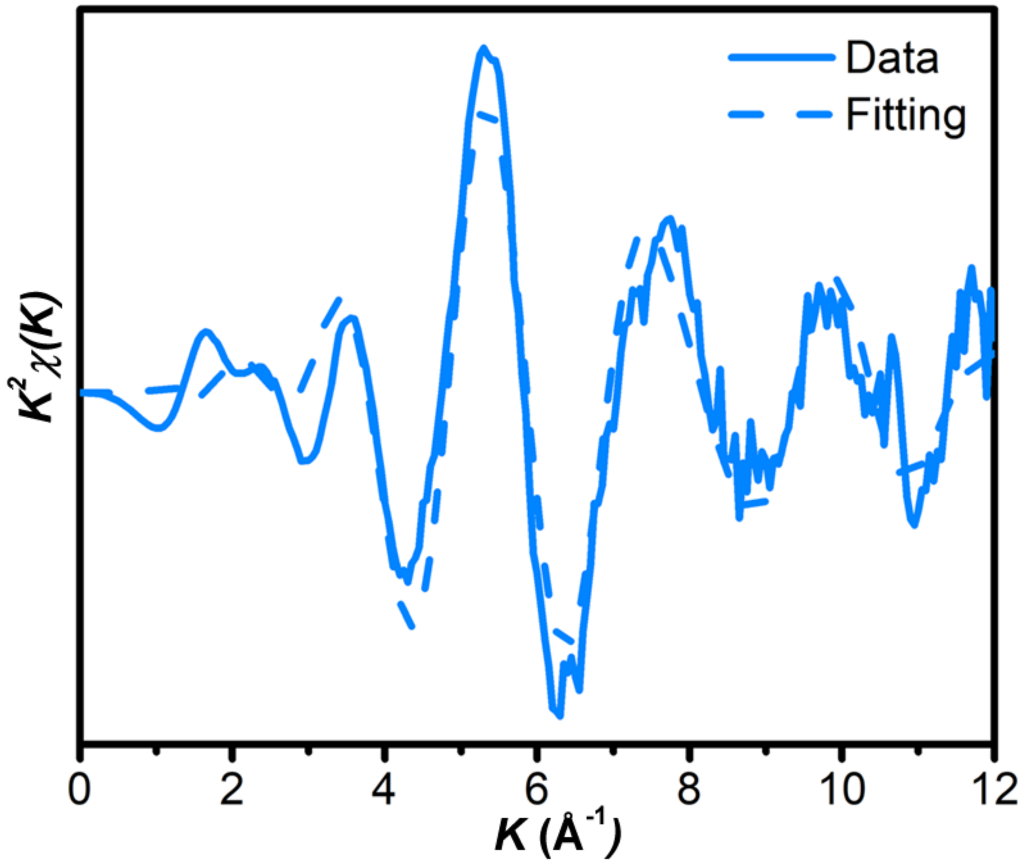

Supplementary Figure 2| Data and fitting result of the k^2-weighted $\chi(k)$ result of EXFAS analysis.

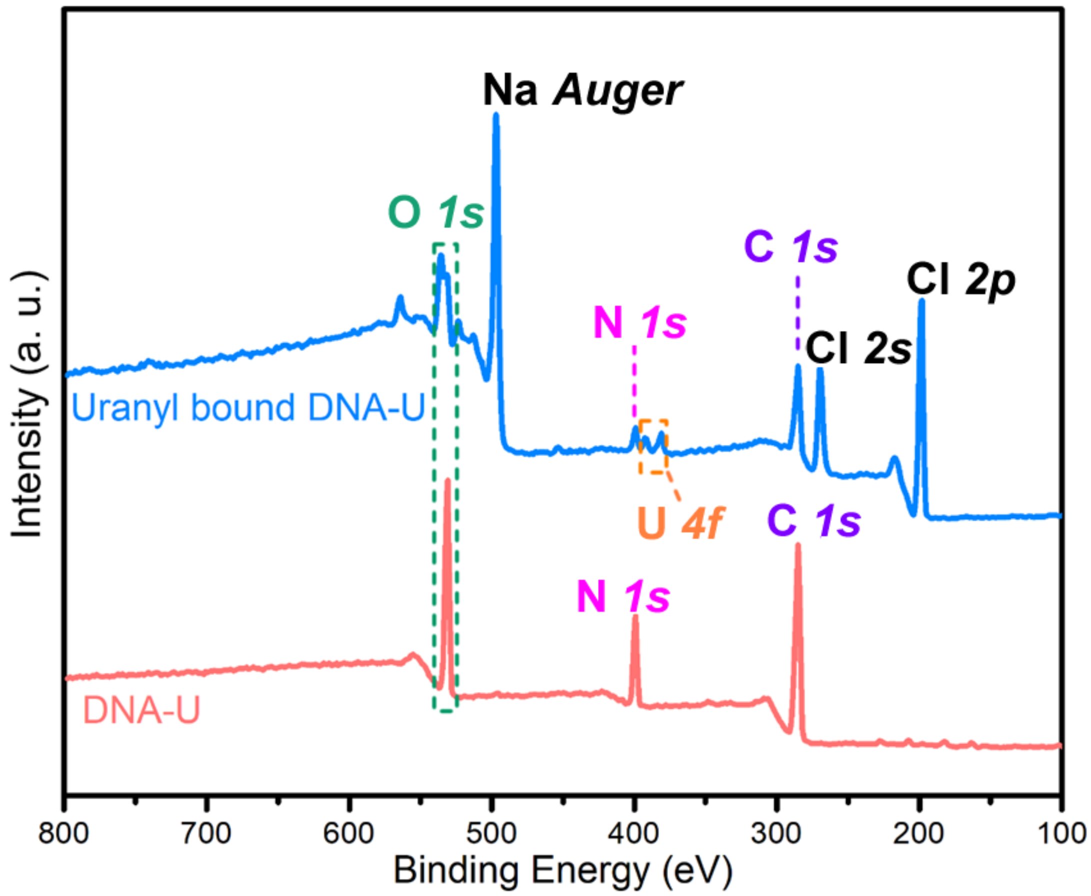

Supplementary Figure 3 XPS analysis of hydrogel DNA-UEH before and after uranyl binding.

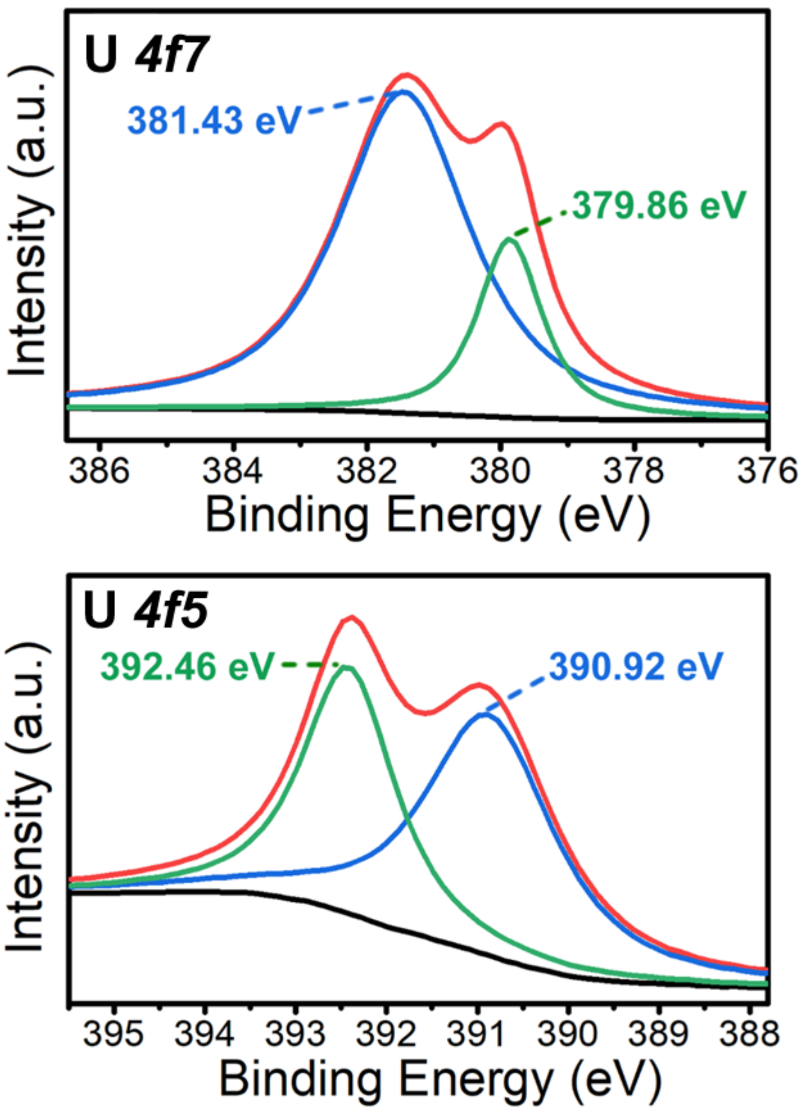

Supplementary Figure 4 High-resolution XPS analysis of uranium element bound by hydrogel DNA-UEH.

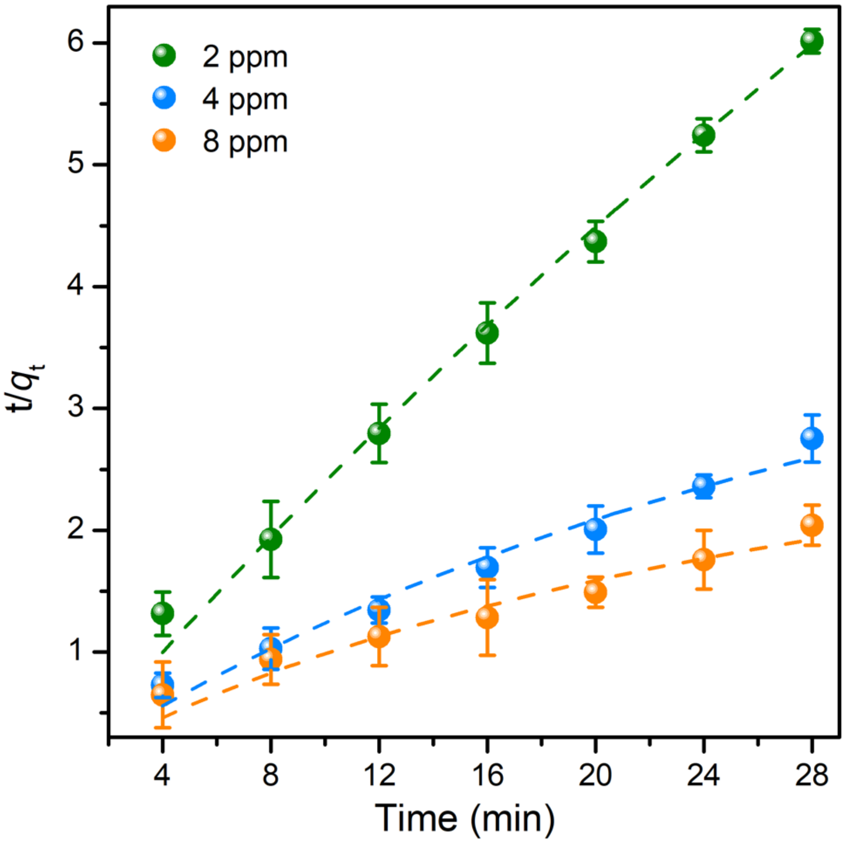

Supplementary Figure 5 The fit of t/q_t to the contact time (t) based on the pseudo-second-order model. The error bars indicate standard deviation (n = 3).

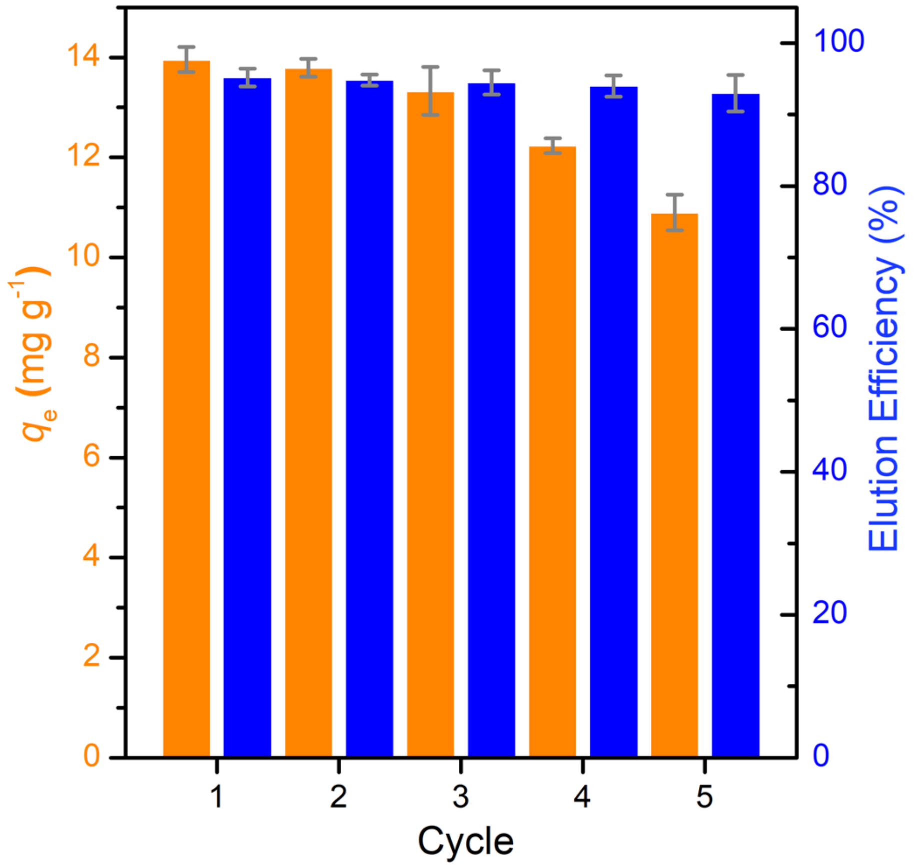

Supplementary Figure 6 Reusability of hydrogel DNA-UEH in 8 ppm uranium spiked simulated seawater. The error bars indicate standard deviation (n = 3).

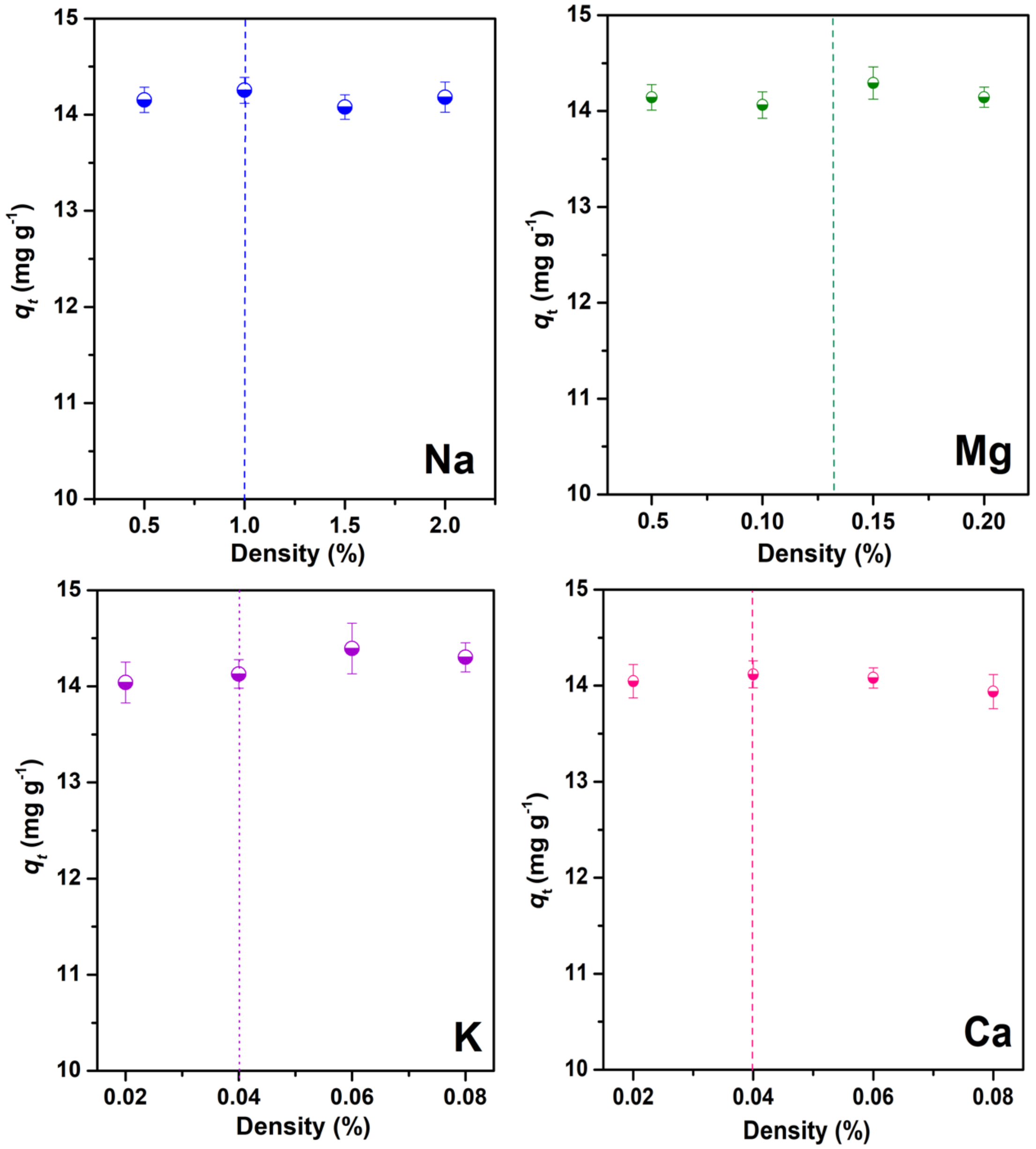

Supplementary Figure 7 Influence of concentrations of interfering metal ions on the uranium adsorption capacity. The uranium solution with a concentration of 8 ppm was used and the concentrations of metal ions in real seawater were shown. The error bars indicate standard deviation (n = 3).

Supplementary Table 1 Oligonucleotide sequences of linear ssDNA and primers used for fabricating of the DNA hydrogel.

Strand	Sequence
**Linear ssDNA**	5'-PhosphateTCGTTTGATGTTCCTAACGTACCACACGTCCATCTCTGCAG TCGGGTAGTTAAACCGACCTTCAGACATAGTGAGTCATAGAGGCATTG GCTG-3'
**Primer1**	5'-TAGGAACATCAAACGACAGCCA-3'
**Primer2**	5'- ACGTACCACACGTCCATCTCT -3'
**Primer3**	5'- ATAGTGAGTCATAGAGGCAT -3'

Supplementary Table 2 Fitting results for EXAFS analysis of uranium bound by hydrogel DNA-UEH.

**path**	**N**	σ^2 ($Å^2$)	**R** (Å)	**R-factor**
$U\text{-}O_{uranyl}$	2.0 ± 0.2	0.0038 ± 0.0012	1.79 ± 0.02	0.024
$U\text{-}O_{phosphate}$	2.2 ± 0.5	0.0038 ± 0.0012	2.24 ± 0.02	0.024
$U\text{-}O_{carbonyl}$	2.6 ± 0.6	0.0038 ± 0.0012	2.40 ± 0.02	0.024

Supplementary Table S3 Concentrations of uranium and co-existing metals in 100 times concentrated seawater.

Co-exiting Metals	Con. In natural SW (ppb)	Con. In 100×SW (ppb)
Fe	1.37	137.35
Cu	0.60	60.30
Co	0.0050	0.50
V	9.71	971.50
U	3.35	335.23
Ni	2.11	211
Ba	27.13	2713.50
Sr	2211	2211.36
Pb	0.0020	0.20
Zn	0.41	40.60

Supplementary Table S4 Concentration of metal ions used in determining the competition with uranyl.

Metals	Excess in seawater	Metal concentration (M)	Competitiveness
Na^{+}	3.53×10^{7}	5	2.43×0^{6}
Mg^{2+}	3.92×10^{6}	2	3.38×10^{6}
K^{+}	7.62×10^{5}	4	2.48×10^{6}
Ca^{2+}	7.68×10^{5}	1	9.25×10^{5}
Fe^{3+}	0.041	0.5	1.13×10^{6}
Co^{2+}	0.0015	0.5	9.91×10^{6}
Sr^{2+}	658	0.5	1.11×10^{6}
Mn^{2+}	0.028	1	1.65×10^{6}
Ba^{2+}	8.11	0.25	5.09×10^{5}
Cr^{2+}	0	0.5	1.11×10^{6}
Cu^{2+}	0.18	0.05	7.10×10^{4}
VO^{2+}	2.896	0.005	9.75×10^{3}
Ni^{2+}	0.63	0.25	4.95×10^{5}
